# Navigating the participatory turn in agricultural and food research: Best practice from citizen science

**DOI:** 10.1007/s13280-025-02151-7

**Published:** 2025-02-21

**Authors:** Raquel Ajates, Petra Benyei, Helen Avery, Egle Butkeviciene, Alexandra Czeglédi, Dominique Desclaux, Gerid Hager, Barbara Heinisch, Peter N. Hoebe, Toos C. G. E. van Noordwijk, Marco Barzman

**Affiliations:** 1 Universidad Nacional de Educación a Distancia, C/Obispo Trejo, nº2, 28040, Madrid, Spain; 2 Instituto de Economía, Geografía y Demografía, Consejo Superior de Investigaciones Científicas, Calle Albasanz 26, 28037 Madrid, Spain; 3 Centre for Environmental and Climate Science, Lund University, Box 117, 221 00 Lund, Sweden; 4 Faculty of Social Sciences, Arts and Humanities, Kaunas University of Technology, A. Mickevičiaus str. 37, 44244 Kaunas, Lithuania; 5 Environmental Social Science Research Group (ESSRG), Bükkszentkereszt, 42. Táncsics Street, Budapest, 3557 Hungary; 6 Institut National de Recherche Pour l’Agriculture, l’Alimentation et l’Environnement (INRAE), Domaine de Melgueil, 34130 Mauguio, France; 7 International Institute for Applied Systems Analysis, Schlossplatz 1, 2361 Laxenburg, Austria; 8 University of Vienna, Porzellangasse 4, 1090 Vienna, Austria; 9 Earthwatch Europe, 102-104 St Aldates, Oxford, OX1 1BT UK; 10 Earthwatch Europe, Zaltbommel, The Netherlands; 11 Wij.Land, Gein-Zuid 23, 1391 JE Abcoude, The Netherlands; 12 Institut National de Recherche Pour l’Agriculture, l’Alimentation et l’Environnement (INRAE), 147 rue de l’Université, 75007 Paris, France

**Keywords:** Agriculture, ECSA principles, Ethics, Food systems, Methodologies, Participation

## Abstract

Food systems have enormous impacts on people and the planet, with agriculture and food research becoming strategic for many countries. However, the way this research is conducted and the rise of new agri-food technologies have ethical and socio-economic implications. To address these, many scholars are gaining interest in participatory methods, such as citizen science, but are unfamiliar with the latest debates on ethical and methodological issues surrounding non-academic stakeholder engagement. In this perspective paper, we revisit the European Citizen Science Association’s (ECSA) Ten Principles of Citizen Science under the specific lens of agri-food research. The discussion presented is based on a review of the state of the art from academic literature, secondary data from agri-food citizen science projects, and the reflections of 11 scientist and practitioners, members of ECSA’s Agri-Food Working Group. The findings reflect theoretical, methodological, and practical implications for navigating the participatory turn in agriculture and food research.

## Introduction: The expansion of citizen science in agriculture and food system research

Agriculture and food systems^1^*agri-food systems* henceforth are key to human and environmental health (Willett et al. 2019), and their significance reaches beyond questions of food security (Mehrabi et al. 2022), or food sovereignty (Levkoe et al. 2019). From the transmission of livestock diseases to humans, to agriculture’s impact on land use, soil health, water, climate and biodiversity (Fanzo and Miachon 2023), as well as issues of land-ownership, public health, or forced migration (Nyantakyi-Frimpong and Bezner Kerr 2017), the agri-food system is inherently transversal to our ecosystems and societies. Moreover, the importance of food for humanity is reflected in the central position these topics have in public and private research and innovation systems, with large amounts of funding being dedicated to agri-food research each year, from seed development to understanding consumer behaviour (Prasad et al. 2023).

Agri-food research entails a specific history of engagement with society. From traditional farmer-led breeding to top-down extension services, and more recently, agri-food citizen science, stakeholder groups beyond professional researchers have been part of this vast area of research for a long time (van de Gevel et al. 2020). The knowledge and localised experience of farmers, agricultural input companies, processors, farm advisers, or consumers has been, throughout the decades, a key contribution to agri-food research, be it through the expertise on growing conditions and methods, food diaries, etc. However, such participation has not always been acknowledged or compensated (Cook et al. 2021). In fact, the widespread market-oriented framing of food as a commodity rather than as a human right has, on many occasions, led to the exploitation of this tacit or informal knowledge to exacerbate corporate control over plant genetic resources or dietary data, severely affecting farmers’ livelihoods, consumer privacy, and increasing corporate power concentration (Ajates 2023). Indeed, recent technological developments, such as new genomic techniques or big data used in consumer behaviour research, raise concerns over how commercial interests and non-public interest could be steering agri-food research (Sumberg 2017). Thus, food production and consumption are not only technical, but political issues, and existing power relations must be taken into account, from corporate food regimes, to former colonial relations and hierarchies of epistemological systems that are ingrained still today in our research practices and institutions (Kimura and Kinchy 2020).

Addressing agri-food research challenges requires systemic, long-term, and transdisciplinary approaches that incorporate diverse stakeholders´ perspectives (Lamine 2018). In this line, Citizen Science (*CS* henceforth) is a research approach that involves citizens in different stages of the research process. When this involvement goes beyond contributive citizen science—which only focuses on enabling the contribution of data—and promotes long-term active involvement in research, CS can be very effective in generating changes in practice and policy, particularly in food and agriculture (Benyei et al. 2023). Numerous studies suggest that engaging society in participatory scientific knowledge generation is likely to increase the effectiveness and efficiency of the research process itself. Participation can empower marginalised social groups and improve environmental sustainability (van de Gevel et al. 2020). However, not all forms of societal engagement in agri-food research devote sufficient attention to inclusiveness, equity, and empowerment of participants. Indeed, some authors suggest that there is a growing trend of “citizen washing” in research and policy-making that appears to involve the public without, in fact, taking their views into account (Oleart 2023). For instance, certain applications of citizen science have been criticised as a utilitarian approach to gather data for free in projects that only involve citizens in data collection (Vohland et al. 2019). Furthermore, research into the participation patterns in CS suggests a participation bias towards older and more educated men (Paleco et al. 2021). Thus, despite the growing and outstanding efforts to produce documentation supporting good practices in scientific communication and engagement (de Vries et al. 2019), substantial efforts are still needed to ensure inclusive and equitable research practices, particularly in agri-food research involving activities and outcomes that are crucial to people’s lives.

With these considerations in mind, in this perspective paper, we propose a guiding framework for participatory agri-food research, with the aim of contributing to a transition towards just, empowering and equitable food systems research. The framework draws on the European Citizen Science Association’s (ECSA) 10 principles for citizen science (ECSA 2015), which we introduce in the next section. We then discuss the theoretical, methodological, and practical implications of the principles, organised in three keystones, to reflect on their meanings, highlighting potential synergies between principles and how they can be applied to agri-food research. We end by providing recommendations for future research.

### ECSA principles guiding framework—three keystones to highlight synergies between principles and maximise uptake

**Fig. 1:**
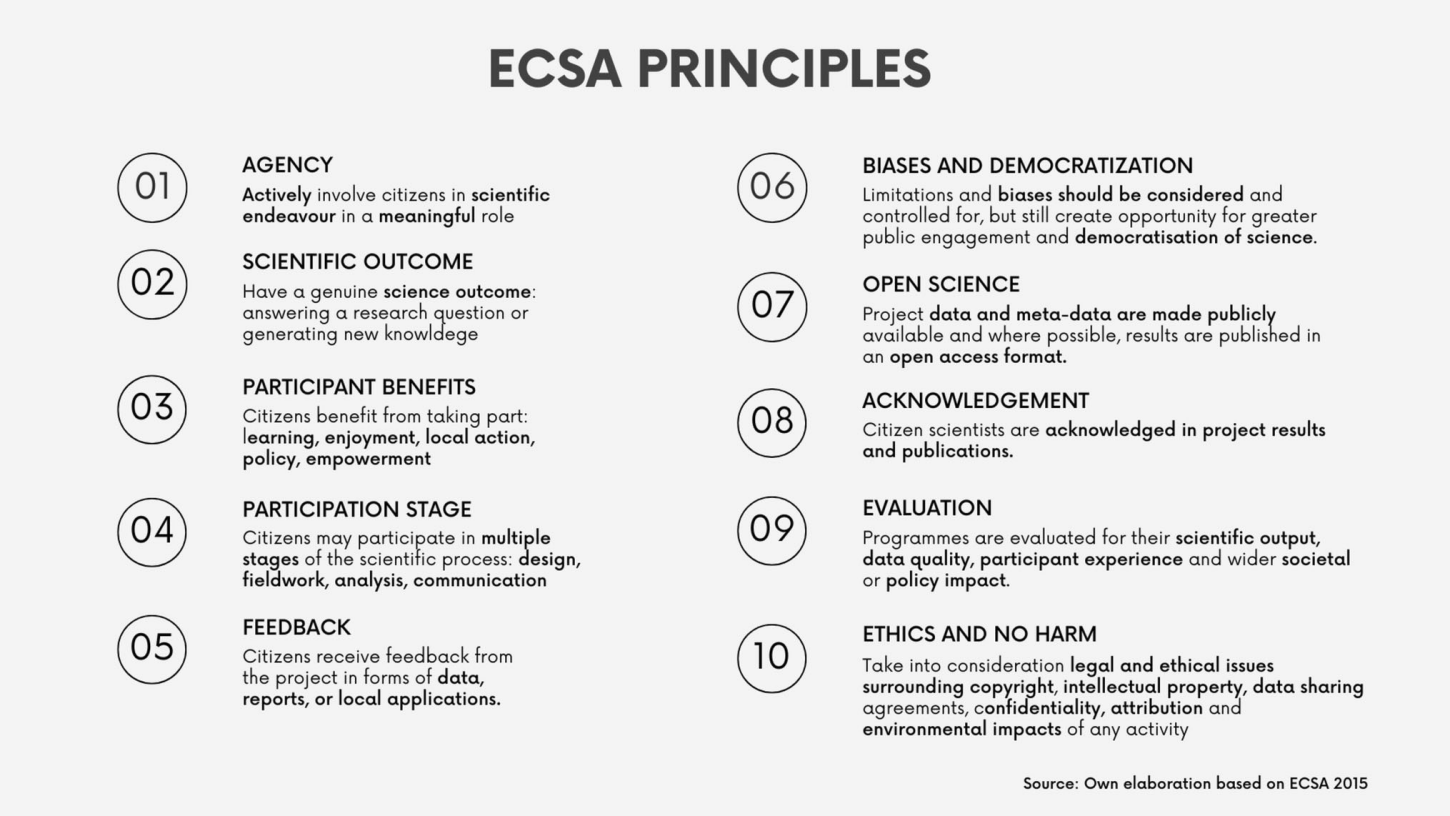
ECSA ten principles of citizen science (2015). Own elaboration using Canva

The ECSA principles for citizen science—illustrated in Fig. 1—were developed by the “Sharing best practice and building capacity” ECSA Working Group in 2015, led by the Natural History Museum of London (ECSA 2015). Communicating these principles with the wider agri-food system research community is becoming crucial, as many scholars and disciplines are gaining interest in participatory and CS methods while unaware of the existing principles that inform these practices (Ryan et al. 2018). Being aware of the principles is also important for funding agencies and evaluators who develop and evaluate agricultural and food systems research funding calls that include a societal engagement priority, as well as for authorities, organisations, and businesses that engage in participatory agri-food research projects or have interest in their outcomes.

**Fig. 2:**
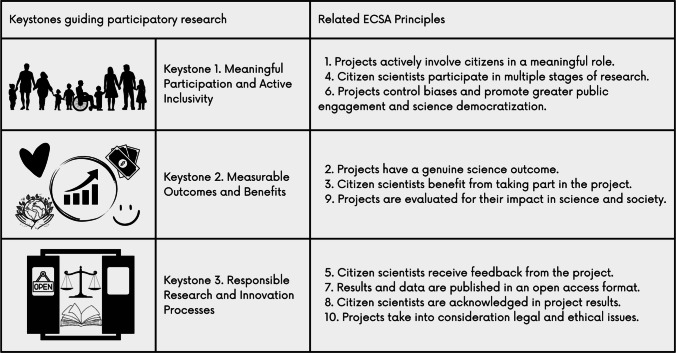
Keystones guiding participatory research and their connection to ECSA principles. Own elaboration using Canva

As outlined above, this paper sets out to re-examine the ECSA principles under the specific lens of agricultural and food system research. This analysis is based on academic literature on agri-food CS, secondary data from agri-food CS projects (such as deliverables, website content, etc.) and the reflections of 11 scientists and practitioners from seven countries who are members of ECSA’s Agri-Food Working Group and who have participated in numerous agri-food CS projects. Additionally, the Agri-Food Working Group held a face-to-face two-day workshop in June 2023, which included an extended discussion and group work to map how the ECSA principles related to and can strengthen CS practices in agri-food research. Small groups were created and assigned one ECSA principle each to be discussed from an agri-food perspective. When the joint output was analysed, we identified similar arguments and themes across several principles, based on which, we put forward the following keystones, summarised in Fig. 2.

Keystone 1. Meaningful participation and active inclusivity, is related to the role of citizens as citizen scientists and the promotion of greater public engagement in science. It includes principles that promote CS inclusivity, active, and meaningful engagement opportunities for citizens, and the acknowledgement and reduction of research biases to support science democratisation. Keystone 2. Measurable Outcomes and Benefits, refers to the expected types of results, their impacts, and the ways to measure such impacts. It includes principles that promote reflexive impacts of CS projects, or the promotion of scientific and non-scientific outcomes and benefits, and the encouragement of evaluation as a tool to measure the multiple effects of projects. Finally, keystone 3. Responsible Research and Innovation Processes, relates to the research process itself in the context of implementing a CS approach. It includes principles that promote responsible processes in CS design and development, including giving meaningful feedback to participants, acknowledging their participation, and following ethical research standards.

We propose the use of these keystones not to replace, but to more easily communicate the ECSA principles to researchers new to CS approaches and keen on adopting them, and for those who use them regularly and would like a briefer framework to apply and share in their practice. The main goal of the piece is to contribute to the discussion on how to better guide participatory agri-food research, and the new keystone framework is used as a mechanism to support this objective, while rooting the discussion in the wider citizen science literature. In the next sections, we discuss how each of these complementary and non-vertical keystones is relevant to agri-food research, in the hope that our work stimulates debate and fosters the implementation of the ECSA principles in this field.

### Keystone 1: Meaningful participation and active inclusivity

Agri-food research has used participatory approaches and engaged citizens, including members of rural communities, for a long time, albeit often in roles with limited acknowledged agency in the research process itself (Davidson 2007). For example, farmers may contribute knowledge and information as part of field trials, surveys, interviews, storytelling, or focus groups, but are not always included in decisions on research questions and methods. Citizen science, in some cases, has also struggled with biased and non-inclusive engagement, limiting its potential impacts by engaging mainly predominantly white, male participants from higher education and affluent socio-economic backgrounds (Allf et al. 2022). This pattern would exclude many agricultural and food service workers from agri-food citizen science. A similar pattern of lower levels of female participation has also been identified in agricultural research (Dey de Pryck and Elias 2023). Several efforts are currently underway to make CS practices more accessible and inclusive for everyone, leading to best practice guidelines and inclusive design methods that promote science democratisation (Benyei et al. 2023; Veeckman et al. 2023), which research in the agri-food domain can benefit from.

From our analysis, three topics emerge as fundamental in this keystone: the agency of participants (practitioners from the food system), the availability of diverse and meaningful project roles, and the reduction of participation barriers and biases. Regarding participants’ agency, the definition of “active” participation is essential. Although the level of participation may vary among projects, it helps to differentiate projects where citizens are “being studied” (traditional social science) from projects where citizens are “those who study” (citizen science). For example, in agri-food research, a focus group might be used as a method to investigate farmers´ choice of crop varieties. However, if attending a focus group is the only role farmers play in the project, lacking any agency on how the research is conducted, it cannot be considered CS, and some would argue that it is not even “participatory” (Cornwall 2008). This keystone and the CS literature also suggest different possible roles for participants, depending on their level of involvement (Haklay et al. 2021), and that every role taken should be meaningful, both to the participant and to achieving the goals of the research activity itself. However, CS participants in agriculture and food research often mostly provide data and are rarely involved in other stages of the research process. This could be due to a prevalence of “short-term” project formats, as deeper engagement levels require more in-depth training, trust, and commitment from citizen scientists (Ebitu et al. 2021). Finally, this keystone also acknowledges the need to democratise science in order to avoid biases. In CS, anyone can take the role of a citizen scientist or co-researcher, independently of a citizens’ legal status, gender, age, education, or expertise. This is particularly significant in the agri-food sector, which employs about 40% of the global workforce, in many cases with poor and illegal working conditions (Davis et al. 2023). Thus, citizen scientists in agri-food can be food consumers as well as agricultural workers and anyone else across the entire food system (Parsons et al. 2019), including those with less power and who might be more transient: migrant farm workers, agricultural students, elderly traditional farmers, female food vendors, food self-reliant indigenous communities, food community groups, food bank users, hobby food growers, food waste composting networks, amongst others. Broadening the diversity of participants’ profiles contributes not only to more equitable research frameworks, but also to a reduction in scientific biases, and thus, improved data quality and relevance (Graham et al. 2014; Nature 2015).

Some key issues must be considered with regard to this keystone. From an implementation viewpoint, it is important to clarify expectations and priorities, including the overall aim and expected outcomes of the project (e.g. pest control monitoring for a farm, on-farm experiments for testing the rollout of a new technology, monitoring societal impact in food deserts, etc.). Also, CS methods and training must be offered in order to foster citizen participation throughout the different research stages. Tailoring such training to the specific context and audience will enable wider participation, remove barriers, and reduce biases. An inclusive training strategy during an agri-food CS project can support citizens moving from collecting data, to analysing them, and even setting up their own bottom-up experiments (Woods et al. 2020b). As research teams grow to incorporate the participation of diverse citizen scientists, their background, interests, and worldviews—as in any professional research team–might affect the steps and decisions in which they are involved within the research process. One hurdle to overcome is the under-valued capacity of citizen scientists to engage in research design, funding procurement or science communication, sometimes due to a lack of self-perceived legitimacy (Benyei et al. 2020a). This is why the process of opening a wider range of project tasks to citizen scientists requires discussions on power sharing, uneven availability of time and resources and differentiating between willingness, motivations, and evolving levels of ability to participate (Cornwall 2008). Also, in the agri-food domain, applying the principles of this keystone involves reverting a long history of colonial farming dynamics that still shape food systems today, from dietary preferences (e.g. tea in UK) to development approaches (e.g. agricultural links with former colonies). Many food practices have emerged in the context of dispossession of indigenous peoples, stratification of food production and distribution based on ethnicity, class and citizenship (Kimura and Kinchy 2020). Furthermore, the power of large corporations on practices and norms in the production, distribution and consumption of food should also be considered.

This keystone of agri-food citizen science can guide research that is more grounded and relevant to the communities involved, also helping to increase their agency. These benefits of strong and diverse stakeholder engagement in research projects have long been recognised (Pateman and West 2023). For example, farmers involved in networks that include researchers, advisers, and other stakeholders are known to be more likely to innovate than isolated ones, and ensure the sustainability of proposed innovations (Barzman et al. 2015). Participants that have a higher level of involvement in projects are also more likely to engage in fund raising and dissemination, thereby fostering increased new participant engagement in subsequent stages (Arnstein 1969). Expanding research teams through CS can contribute to democratising the generation of scientific knowledge (Pearce 2010), resulting in greater public engagement with the potential to disrupt the dominant agri-food system, challenging power dynamics, particularly when involving previously marginalised participants.

### Keystone 2: Measurable outcomes and benefits

Pateman and West (2023) and Van Noordwijk et al. (2021) have notably identified various impact pathways for CS projects, while Wehn et al. (2021) have developed principles for a consolidated approach to impact assessment in CS. Moreover, in the agri-food domain in particular, a number of studies have highlighted the importance of CS in generating scientific, practice, and policy impacts (Ryan et al. 2018; van de Gevel et al. 2020). Still, agri-food research has traditionally followed an approach to project evaluation that values exclusively research and innovation outputs (Turner et al. 2022). Thus, the outcomes and benefits of participatory approaches to agri-food research need to go further, as there are many aspects of participation and areas of the food system that are not always considered in impact assessment (Mourad et al. 2020; Reynolds et al. 2021).

This second keystone highlights three main ideas based on the ECSA principles it encompasses. First that, while having a scientific outcome (mandatory in order to be called “science”), CS should also aim to have impacts beyond the traditional scientific realm, into the educational, economic, socio-cultural, and policy spheres. Second, that such impacts and outcomes should be evaluated based on a diversity of social, political, and scientific indicators. Third, that benefits for participants might be different than those envisioned by scientists. While citizen science must be a research activity and thus provide scientific outcomes in any of the formats the multiple scientific disciplines foresee (from natural sciences, to social sciences and humanities), an exclusive emphasis on scientific outcomes may deter the engagement of stakeholders from diverse backgrounds, or constitute barriers for other types of knowledge beyond academic knowledge to emerge (Benyei et al. 2020a). Indeed, in agri-food CS it is common that the subject of research is directly linked to participants’ business and economic situation, e.g. farmers contributing to the investigation of farmland soil health, which can have direct effects on yield and hence, farmers’ income. Thus, citizen scientists’ motivations to participate in an agri-food project, and their expectations in relation to project outcomes and impact might go beyond the ones that are written in the project proposal (Ryan et al. 2018). For example, NGOs or local residents might initiate agri-food CS projects to raise awareness of social injustices such as land grabs or food deserts, or to stop damaging farming practices by collecting impact indicators in affected ecosystems, or, map illegal deforestation and land uses (Kimura and Kinchy 2020). Considering this and given the multi-actor, multi-output, and transformative character of participatory agri-food research, including more-than-academic outcomes in evaluation become crucial. Moreover, to avoid exploiting citizen scientists’ time and goodwill, feasible and tailored rewards and benefits must be considered collectively from the beginning of the project. In the case of agri-food CS, this reward could be improved practices on pest control, water management, or soil management (Ryan et al. 2018; Woods et al. 2020b) that could translate into health outputs from reduced pesticide use, savings and self-pride, or other less tangible benefits, such as improved interactions and learning from fellow farmers or citizens (Benyei et al. 2020b). Indeed, the different stakeholders can benefit from gaining ownership and agency over project results, providing them with the evidence and the skills that can empower them to take further actions and contribute to broader social movements (Benyei et al. 2023).

This keystone encourages researchers to consider the potential tensions between academics and citizen scientists in relation to possibly different views on the expected project goals to be evaluated, and the challenges of involving stakeholders beyond academia in project evaluation as well as delivering evaluation reports that are meaningful and timely, both to funders and participants. These tensions arising from differing constraints and outcome priorities of academic and non-academic participants, are issues extensively discussed in the literature (Eitzel et al. 2017; Ebitu et al. 2021). Also, during the data collection phase, conflicts may arise between scientific goals and participants’ food and farming practices, e.g. projects investigating sustainable agricultural practices in a region characterised by intensive farming. Thus, methodologically, co-created and validated data collection protocols and tools will enhance the knowledge transfer and implementation of project outcomes, and their credibility and acceptability by different stakeholder groups. It is key to realise that such collaborations have the potential to not only change citizen scientists’ perceptions, but also broaden scientists’ understanding and perception of other professionals involved, with a potentially profound impact, e.g. on their future research priorities (Benyei et al. 2023). Furthermore, involving citizens in evaluation can be challenging, and the team must ensure a safe space to encourage participants to provide constructive feedback in suitable formats, on things that went well, as well as areas to improve. Evaluation methods might encounter differences not only in spatial and temporal horizons considered, but also in terms of the weight attributed to the various aspects being evaluated. Disciplinary, social, and professional hierarchies within teams can be an issue when establishing these priorities and also when setting data quality standards (Sumberg et al. 2013). Indeed, measuring data quality in participatory projects presents several challenges (Steinke and van Etten 2017). Thus, agreement on terminology must be considered carefully (Eitzel et al. 2017). This is particularly important in agri-food research, where a multitude of tools, definitions, and practices exist for capturing “social impact” (Janker and Mann 2020). Similarly, many other key concepts in agri-food research lack an agreed definition and/or operationalisation (e.g. sustainable and healthy diets, local food, or even healthy soil) mainly due to the high number of factors determining each of these constructs. In addition, evaluation timing and methods pose important considerations, and while evaluation is often thought to take place at the end of a project, impact targets should be predefined together with participants from the beginning of the collaboration (Wehn et al. 2021). Finally, the involvement of relevant policy makers from the beginning of the project, e.g. through the creation of observatory policy interfaces or liaising with national statistics units, can help link the project outcomes with policy frameworks such as the Common Agricultural Policy (CAP), local farming schemes, rural repopulation programmes, and others (Woods et al. 2020b). More specifically, due to its complex links of natural and social dimensions, a global food policy area to which agri-food CS is contributing, is through measuring SDG indicators related to water, soil, zero hunger, good health, life on land, and below water, etc. (Ajates et al. 2020; Fraisl et al. 2020).

Agri-food CS is well placed to have outcomes that span from the hyperlocal to the global and that can be demonstrated through a more comprehensive evaluation (Ajates et al. 2020). Tools for co-designing and co-evaluating impacts of agri-food CS projects exist and can be adapted to different project needs (Woods et al. 2020a). Thus, this keystone maximises the reach of project documented and multilevel impacts that can promote the funding and use of participatory approaches in agri-food research and beyond.

### Keystone 3: Responsible research and innovation processes (RRI)

Discussion about RRI in agri-food research and development is increasingly popular, especially in the field of agricultural digitalisation (Bellon-Maurel et al. 2022). Tricarico et al. (2020) have highlighted four RRI dimensions that must be examined: (1) The processes of mutual exchange in setting and re-driving the direction of research and innovation (Diversity & Inclusiveness); (2) socially desirable science and innovation (Anticipation); (3) participatory and accessible methodologies experimented in the research agenda and the dissemination of its outcomes (Openness and Transparency); (4) flexible, reflexive, and socially responsible governance of the process (Responsiveness and Adaptation to Change). However, a key differentiating aspect of CS approaches with regard to RRI is the active role of societal actors in research, as we discussed in Keystone 1.

Keystone 3, while being in dialogue with the RRI approach, incorporates the specificities of CS, where two main topics are fundamental: the ways in which results and data are shared with the citizen scientists involved in the project and more broadly with wider society, and the ethical principles CS is set to follow. Facilitating access to results and data and providing project feedback to participants in a suitable way are two crucial separate but intertwined elements of this keystone. While providing feedback to participants can be seen as an essential aspect contributing to the responsible governance of the research process, making results and data accessible contributes to a broader openness and transparency goal (Woods et al. 2020b; Bellon-Maurel et al. 2022). Feedback can be more specifically tailored to the needs or expectations of participants, while making research outputs accessible should follow guidance from the wider movement of scientific openness and institutional policies on open data (Wehn et al. 2024). ECSA and the Association for Advancing the Participatory Sciences (formerly known as the US CS Association) have highlighted strong synergies between CS and open science movements, with specific working groups dedicated to this topic (see ECSA and AAPS websites^2^). The growing number of CS projects adhering to this movement in health and genetic research projects (DITOs Consortium 2017) can serve as inspiration and guidance in CS agri-food research. With the rise of the genetic sequencing of plants, fungi, and animals used in farming and food processing, this movement sets to promote open data, open code, and participatory processes that benefit communities and avoid biopiracy (Calvet-Mir et al. 2018; Ajates 2023). Indeed, opening data is one of the key differentiating aspects that CS brings to participatory agri-food research, as in many cases, data collected through the latter were kept within the projects, and participants’ knowledge was considered—just as seeds or plants—another resource that could be appropriated and capitalised (Birch et al. 2010). Regarding ethical principles, this keystone highlights the need to follow ethical guidelines and consider the regulatory framework that comes with the benefit-sharing principle and no-harm rule of any scientific research. Ethical issues such as data sovereignty or human rights protection in agri-food CS have been discussed before by CS practitioners (Reyes-Garciá et al. 2022; Benyei et al. 2023). Although, in principle, most agri-food participatory research has to be approved by institutional ethical review boards, the literature and this keystone remind us that ethics go beyond ticking pre-defined boxes and should engage more deeply into—sometimes, uncomfortable—discussions between researchers and participants about power sharing and participants’ rights. A specific aspect of ethics covered in this keystone is attribution. Research within the agri-food sector has increasingly acknowledged the significance of involving stakeholders, encompassing farmers and agri-food businesses, in the outcomes and publications of projects (Jones et al. 2014). The attribution of communities collectively credited in the acknowledgement section of journal articles is a common manifestation of participatory research ethics. However, defining what acknowledgement entails for individual participants, requires joint consideration. Acknowledging research participants is centred on participatory research ethics that require the perception of citizen scientists as active contributors. This is further emphasised by processes of knowledge co-production (Pearce 2010). Without trustable collaborations with participants, acknowledgement might remain an ethically motivated, but merely symbolic gesture. This keystone opens the floor to discussing how a dynamic view of acknowledgement, involving participants in the co-definition of what is expected, could be a better approach, potentially leading to alternative forms of publication, outputs, and enhanced engagement.

With regard to adequate levels of feedback, this third keystone notes areas for agreement. First, establishing what appropriate feedback is might become challenging when working with multiple stakeholders. As noted by De Vries et al. (2019), in general “little research has thoroughly investigated the extent to which citizen scientists find communication of scientific output to be important” (pp.1), and even less in the area of agri-food. Moreover, even in participatory projects, feedback and access to data/results may not be relevant for participants, especially when limited to websites, scientific reports, or raw data, failing to impact participants’ practices. For instance, in the FARM project,^3^ when asked about what the agricultural students expected from the project, most students did not mention feedback nor data. To tailor feedback and results/data sharing appropriately, and considering that feedback provision has a positive impact on participants’ motivation to further engage in CS (Cappa et al. 2020), a feedback form or community engagement protocol should be established at the start of a CS project and be monitored throughout its implementation. This protocol needs to draw on early discussions on project output criteria (see Keystone 2). It should also include relevant timing, content, and form of feedback. Moreover, it should consider to which extent the feedback should be open to everyone or available and tailored to certain project partners, as this might have ethical, financial, or legal consequences. Indeed, a second related issue central to this keystone refers to data privacy. Opening data might be particularly impactful for agri-food citizen scientists, as data sensitivity, particularly concerning farmland or consumer choices, may directly affect farmers’ livelihoods and business interests, and consumers’ privacy. For instance, discovering protected species or flood risk could lead to land designation and/or land value changes, or finding negative environmental impacts could further polarise farmer-environmentalist discussions (Burton et al. 2008). In other cases, participants in CS projects addressing health or environmental impacts of industrial food supply chains might even face intimidation by industry stakeholders, or the research results might be put under special scrutiny for being a CS project (Kimura and Kinchy 2020), especially if these projects challenge the existing norms of dominant agricultural practices.

Moreover, potential data use by third parties, such as big companies modelling food markets, raises concerns about consumers’ information misuse and lack of protection. This is why a set of ethical principles should always be implemented in parallel. As agri-food research deals with peoples’ basis for survival (food) or income (farming), extra ethical and legal considerations might be necessary. For instance, the issue of patents in agri-food participatory research requires early and deep consideration given its potential legal, ethical, and economic impacts (Fredriksson 2021). Some projects choose not to patent or favour other forms of licencing (e.g. copy-left), and it is common to encounter challenges when private companies exploit shared knowledge without proper compensation to the less privileged parts involved (Calvet-Mir et al. 2018). Indeed, biopiracy is a significant concern in participatory agri-food research, with considerable impacts in terms of community’s food sovereignty and access rights to natural resources (Ajates 2023). To help manage these issue, CS projects must establish a data sharing protocol that is agreed upon with participants before sharing any data, and ideally, before data collection has started, to make the project as open as possible and as closed as necessary. Thus, it is key to identify and deal with any potential privacy concerns before collecting and opening the data. Robust data management strategies will consider different types of outcomes, how they are going to be shared, and who will likely benefit from them. Moreover, it is important to remember that access does not equal usability; thus, user requirements and skills must be taken into account in data sharing policies. A third issue relates to the multiple and sometimes conflicting ways in which recognition and benefit sharing materialises in projects. A big discussion has been ongoing in the CS community about participant economic compensation, the role this might have in promoting more participant diversity and the impacts it can also have in terms of the relationship between participants and researchers (Benyei et al. 2023). For instance, in agri-food CS, paying farmers for their time and effort is starting to be a common practice (see RADIANT project,^4^), although it can create conflicts when resources are limited and not all farmers can participate. In a situation with numerous contributors or groups, rewards and recognition are better preceded by a consultation and alignment of expectations. The symbolic recognition of contributions such as co-authorship, active recognition with delegated responsibilities such as co-writing, co-organising and co-chairing research tasks and workshops, community champion roles (Woods et al. 2020b), offer different strategies for appreciating participants’ contributions. Moreover, an advocacy lens can empower communities, leveraging diverse forms of acknowledgement for change and community organisation, which is especially relevant for agricultural cooperative endeavours in specific geographical areas.

In sum, co-decisions on the most appropriate form of acknowledgement and rewards, data, and result sharing and feedback are necessary with farmers, consumers, and other stakeholders to ensure their ongoing commitment to the research. This keystone sets the field for more ethical, just, and equitable agri-food research practices and minimises the risks of projects having a negative impact on the communities they work with.

## Conclusions and way forward

Agri-food research is shaped by the myriad of disciplinary lenses and methods it applies, and the far-reaching implications of food in society. The food system is composed of complex socio-ecological systems that are often characterised by policy, technological, environmental, and social aspects that require a transdisciplinary approach (Lamine 2018). Citizen science in agri-food opens up a common umbrella under which diverse participatory scientific activities and methods—such as farmer-based biodiversity monitoring, participatory plant breeding, rapid rural appraisal, food deserts mapping, etc.—can be in dialogue and create wider public engagement in addressing urgent food system challenges.

Agri-food research is currently faced with accelerating socio-technical changes and environmental concerns that complicate ethical considerations within this research landscape. In this context, we have underscored the need for guidelines that contribute to an inclusive, equitable, and empowering implementation of agri-food research working with and for society. Building on the ECSA principles we have provided three keystones offering such guidance on how to involve participants in agri-food research in an ethical and practical manner. This new framing of the ECSA principles should facilitate their implementation by researchers not yet involved in the CS community, but interested in applying participatory methods, or those looking for a more integrated version of the principles.

The CS approach highlights how ethics, rewards, and data usability are crucial in participatory agri-food research. At the same time, an agri-food system lens enables the CS community to move beyond environmental impact criteria towards a more integrated understanding of the social, economic, and public health impacts of their research (Wehn et al. 2021).

This perspective piece has opened up many questions that can inform future research and practice, and has proposed a framework that can be applied to specific agri-food CS established and emerging domains. We have highlighted the widespread level of impacts that agri-food CS can achieve, since it examines food as a life-dependent daily practice and livelihood relating to most of the SDGs, representing a collective drive for positive change, rooted in respect, openness, and empowerment.

The reflections provided in this perspective article are based on transdisciplinary scholarly literature and our diverse work in agri-food CS and participatory science. However, we envision this framework to be communicated beyond the European Citizen Science Association networks to reach and be taken up by all agri-food researchers. We also think it could be interesting to funding agencies and evaluators who develop and evaluate agricultural and food systems research funding calls that include a societal engagement priority, as well as for authorities, organisations, and businesses that engage in participatory agri-food research projects or have interest in their outcomes. We have aimed to provide food for thought and encouragement on how agri-food research can draw from CS principles at the intersection of food security, ethics, and technology debates. Our hope is that through such integration of research domains, knowledge generation can be made more equitable, locally relevant, and engaging.

## Footnotes

1 We adopt a systemic approach to food systems, i.e. take into account all processes and actors involved in food production, processing, distribution, trading, catering, consumption, disposal, etc. (Parsons et al. 2019).

2 https://www.ecsa.ngo/working-groups/citizen-science-and-open-science/ and https://www.citizenscience.org/association/about/values.

3 www.farm.csic.es.

4 https://www.radiantproject.eu/.
